# Algorithms for the quantitative Lock/Key model of cytoplasmic incompatibility

**DOI:** 10.1186/s13015-020-00174-1

**Published:** 2020-07-22

**Authors:** Tiziana Calamoneri, Mattia Gastaldello, Arnaud Mary, Marie-France Sagot, Blerina Sinaimeri

**Affiliations:** 1 Inria Grenoble, 655, Avenue de l’Europe, 38334 Montbonnot, France; 2 Université de Lyon, Université Lyon 1, CNRS, Laboratoire de Biométrie et Biologie Evolutive UMR 5558, 43, Boulevard du 11 Novembre 1918, 69622 Villeurbanne, France; 3 Department of Computer Science, Sapienza University of Rome, viale Regina Elena 295, 00161 Rome, Italy

**Keywords:** Cytoplasmic incompatibility, Chain subgraph cover problem, Enumeration algorithms, Exact exponential algorithms, Interval order

## Abstract

Cytoplasmic incompatibility (CI) relates to the manipulation by the parasite *Wolbachia* of its host reproduction. Despite its widespread occurrence, the molecular basis of CI remains unclear and theoretical models have been proposed to understand the phenomenon. We consider in this paper the quantitative Lock-Key model which currently represents a good hypothesis that is consistent with the data available. CI is in this case modelled as the problem of covering the edges of a bipartite graph with the minimum number of chain subgraphs. This problem is already known to be NP-hard, and we provide an exponential algorithm with a non trivial complexity. It is frequent that depending on the dataset, there may be many optimal solutions which can be biologically quite different among them. To rely on a single optimal solution may therefore be problematic. To this purpose, we address the problem of enumerating (listing) all minimal chain subgraph covers of a bipartite graph and show that it can be solved in quasi-polynomial time. Interestingly, in order to solve the above problems, we considered also the problem of enumerating all the maximal chain subgraphs of a bipartite graph and improved on the current results in the literature for the latter. Finally, to demonstrate the usefulness of our methods we show an application on a real dataset.

## Introduction

**Fig. 1:**
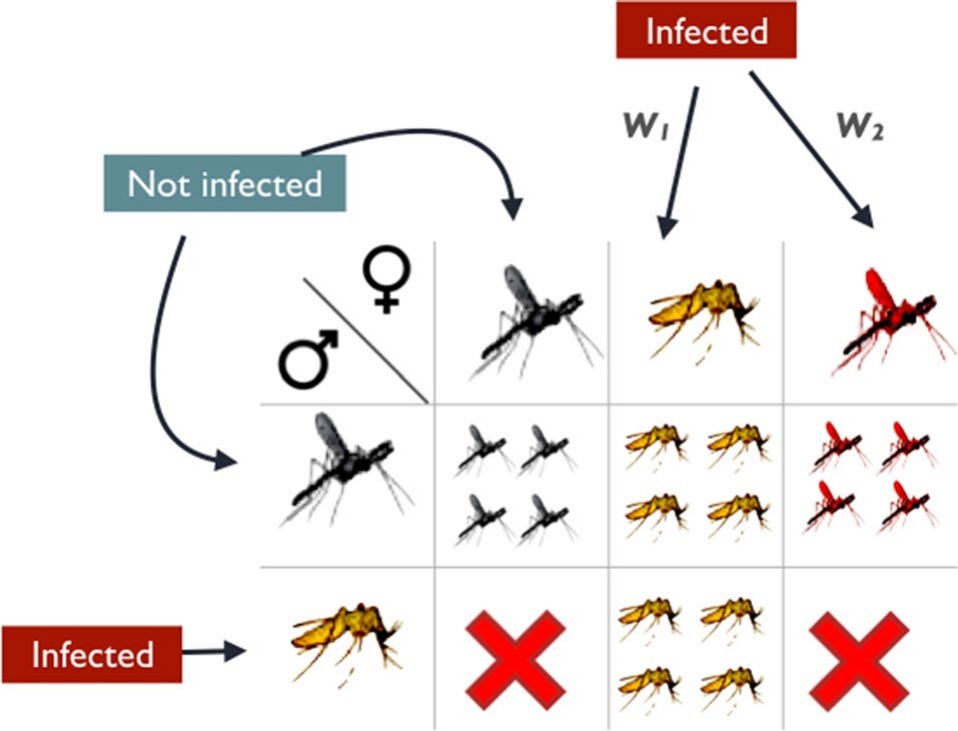
CI of Wolbachia. An example showing the behavior of cytoplasmic incompatibility. The incompatibility occurs not only between infected males and uninfected females but also between males and females carrying different strains of *Wolbachia* (for example strains $w_1$ and $w_2$ in the figure

*Wolbachia* is an intracellular bacterium that infects numerous arthropod species. It is transmitted vertically through the host’s eggs and is known for frequently influencing the reproductive development and behaviour of its host. In particular, the transmission of *Wolbachia* is promoted via a mechanism known as cytoplasmic incompatibility (CI). CI occurs when a *Wolbachia* infected male host crosses with a female that is either uninfected, or is infected by another *Wolbachia* strain. In this case, the cross is unsuccessful and the offspring does not survive. In this way, CI gives a reproductive advantage to the infected females by reducing the reproductive success of uninfected females (for a review on this phenomenon, see for example [1]). An example illustrating CI is provided in Fig. 1. It is a mechanism induced not only by *Wolbachia* but it is also observed in other unrelated bacteria such as for example *Cardinium hertigii* [2, 3]. CI has attracted much attention also for its potential use in biological control, i.e. the introduction of parasites, predators, and pathogens with the purpose to reduce or suppress pest populations [4].

Despite the widespread occurrence of CI, its molecular basis remains unclear and theoretical models have been proposed to understand the phenomenon. The general model assumes the existence of a toxin, deposited by the bacterium in the sperm, which leads to the death of the zygote unless it is neutralised by an antitoxin deposited by the bacteria present in the egg [5]. A more concrete model is the quantitative Lock-Key model which assumes that the toxin and antitoxin are distinct molecules and that the success of the cross depends on the quantity of each of them present in the eggs and sperm (see for example [6]). This model currently represents the best hypothesis and is consistent with the data available (see e.g. [7, 8]).

In [6, 9], the cytoplasmic compatibility relationships that are observed in a given dataset are modelled as a bipartite graph with males and females in different partitions of the graph and edges representing an unsuccessful crossing. The aim is to find the minimum number of different Lock/Key molecules that explain the observed data. This is modelled as finding the minimum number of chain subgraphs (i.e. graphs that do not contain a $2K_2$ as induced subgraph) that cover the edges of a given bipartite graph [6, 9]. Moreover, as different minimum (resp. minimal) covers may correspond to solutions that differ in terms of their biological interpretation, the capacity to enumerate all such minimal chain covers becomes crucial. More formally, in this paper, we address the problem of enumerating without repetitions all maximal *edge induced* chain subgraphs of a bipartite graph. If there is no ambiguity, from now on we will refer to them simply as *chain subgraphs*, omitting the wording “edge induced”.

The problem of enumerating in bipartite graphs all subgraphs with certain properties has already been considered in the literature. These concern for instance maximal bicliques for which polynomial delay enumeration algorithms in bipartite [10, 11] as well as in general graphs [11, 12] were provided. In the case of maximal node *induced* chain subgraphs, their enumeration can be done in total polynomial time as it can be reduced to the enumeration of a particular case of the minimal hitting set problem [13] (where the sets in the family are of cardinality 4). However, the existence of a polynomial delay algorithm for this problem remains open. We recall that an enumeration algorithm is said to be *output polynomial* or *total polynomial* if the total running time is polynomial in the size of the input and the output. It is said to be *polynomial delay* if the time between the output of any solution and the next one is bounded by a polynomial function of the input size [14].

Regarding the problem of enumerating maximal *edge induced* chain subgraphs in bipartite graphs, in [15] the authors deal with it in the form of enumerating minimal interval order extension of interval orders (see “Chain graphs and interval orders” section for the relation between these two problems). In this paper, we improve this result by proposing a polynomial space and polynomial delay algorithm to enumerate all maximal chain subgraphs of a bipartite graph. We also provide an analysis of the time complexity of this algorithm in terms of the input size. In order to do this, we prove some upper bounds on the maximum number of maximal chain subgraphs of a bipartite graph *G* with *n* nodes and *m* edges. This is also of intrinsic interest as combinatorial bounds on the maximum number of specific subgraphs in a graph are difficult to obtain and have received a lot of attention (see for e.g. [16, 17]).

We then address a second related problem called the *minimum chain subgraph cover* problem that, for a given graph *G*, asks to determine the minimum number of chain subgraphs that cover all the edges of *G*. This has already been investigated in the literature as it is related to other well-known problems such as the maximum induced matching (see e.g. [18, 19]). For bipartite graphs, the minimum chain subgraph cover problem is shown to be NP-hard [20]; it is also known that for some special subclasses of bipartite graphs (e.g. convex bipartite graphs or chordal bipartite graphs), the problem can be solved in polynomial time in the size of the graph [19, 21]. Nevertheless, bipartite graphs that represent cytoplasmatic incompatibility in general are neither convex nor chordal (see for example the graph defined by the incompatibility matrix in [6]). Moreover, to the best of our knowledge, no special structural properties are known on CI bipartite graphs and hence we cannot apply these types of results.

Calling *m* the number of edges in the graph, we provide an exact exponential algorithm which runs in time $O^*((2+\varepsilon )^{m})$ (by $O^*$, we denote the standard big *O* notation but omitting polynomial factors) by combining our results on the enumeration of maximal chain subgraphs with the inclusion-exclusion technique [22]. Notice that, since a chain subgraph cover is a family of subsets of edges, the existence of an algorithm whose complexity is close to $2^{m}$ is not obvious. Indeed, the basic search space would have size $2^{2^{m}}$, which corresponds to all families of subsets of edges of a graph on *m* edges.

Finally, we approach the problem of enumerating all minimal covers by chain subgraphs. To this purpose, we provide a total quasi-polynomial time algorithm to enumerate all *minimal* covers by maximal chain subgraphs of a bipartite graph. To do so, we prove that this can be polynomially reduced to the enumeration of the minimal set covers of a hypergraph.

To show the usefulness of our algorithms, we implemented Algorithm 1 and applied it to the *Culex pipiens* dataset [6, 9]. We show that our method allows to identify solutions that are better than the ones presented in the literature, in the sense that they require less Lock and Key molecules to explain the data.

The remainder of the paper is organised as follows. In “Preliminaries” section, we give some definitions and preliminary results that will be used throughout the paper. In “Modeling cytoplasmic incompatibility” section, we better explain the CI-model in terms of a graph problem. “Enumerating all maximal chain subgraphs” section provides a polynomial delay algorithm to enumerate all maximal chain subgraphs in a bipartite graph *G* and “Upper bounds on the number of maximal chain subgraphs” section presents an upper bound on their maximum number. We use the latter result to further establish the input-sensitive complexity of the enumeration algorithm. In “Minimum chain subgraph cover” section, we detail the exact algorithm for finding the minimum size of a minimum chain cover in bipartite graphs, and in “Enumeration of minimal chain subgraph covers” section we exploit the connection of this problem with the minimal set cover of a hypergraph to show that it is possible to enumerate in quasi-polynomial time all minimal covers by maximal chain subgraphs of a bipartite graph. “Chain graphs and interval orders” section deals with the interpretation of the results in “Enumerating all maximal chain subgraphs” and Minimum chain subgraph cover sections in the context of poset and interval poset dimension, two problems which are deeply related. In “A case study” section we show an application of our method to a real dataset. Finally, we conclude with some open problems in “Conclusions and open problems” section.

## Preliminaries

Throughout the paper, we assume that the reader is familiar with the standard graph terminology, as contained for instance in [23]. We consider finite undirected graphs without loops or multiple edges. For each of the graph problems in this paper, we let *n* denote the number of nodes and *m* the number of edges of the input graph.

Given a bipartite graph $G=(U \cup W, E)$ and a node $u \in U$, we denote by $N_G(u)$ the set of nodes adjacent to *u* in *G* and by $E_G(u)$ the *set of edges incident to u in G*. Moreover, given $U' \subseteq U$ and $W' \subseteq W$, we denote by $G[U',W']$ the *subgraph of G induced by *$U' \cup W'$. A node $u \in U$ such that $N_G(u)=W$ is called a *universal node*.

A bipartite graph is a *chain graph* if it does not contain a $2K_2$ as an induced subgraph. Equivalently, a bipartite graph is a chain graph if and only if for each two nodes $v_1$ and $v_2$ both in *U* (resp. in *W*), it holds that either $N_G(v_1) \subseteq N_G(v_2)$ or $N_G(v_2) \subseteq N_G(v_1)$. Note that this means that the nodes of *U* (resp. of *W*) can be linearly ordered, say $v_1, \ldots , v_n$, such that $N_G(v_1) \subseteq N_G(v_2)\subseteq \ldots \subseteq N_G(v_n)$. Given a chain subgraph $C=(X \cup Y, F)$ of *G*, we say that a permutation $\pi$ of the nodes of *U* is a *neighbourhood ordering* of *C* if $N_{C}(u_{\pi (1)})\subseteq N_{C}(u_{\pi (2)})\subseteq \ldots \subseteq N_{C}(u_{\pi (|U|)})$. Observe that if $X \subset U$, the sets $N_{C}(u_{\pi (1)}),\ldots , N_{C}(u_{\pi (l)})$ for some integer $l\le |U|$ may be empty and, in case *C* is connected, $l= |U| - |X|$. By the *largest neighbourhood of C*, we mean the neighbourhood of a node *x* in *X* for which the set $N_C(x)\subseteq Y$ has maximum cardinality. A set $Y' \subseteq Y$ is a *maximal neighborhood* of *G* if there exists $x \in X$ such that $N_G(x)=Y'$ and there does not exist a node $x' \in X$ such that $N_G(x) \subset N_G(x')$. Two nodes $x, x'$ such that $N_C(x)=N_C(x')$ are called *twins*.

In this paper, we always consider *edge induced* chain subgraphs of a graph *G*. Hence, here a chain subgraph *C* of *G* is identified with its edges $E(C)\subseteq E(G)$ while its set of nodes will be constituted by all the nodes of *G* incident to at least one edge in *C*. Since the edges of a chain graph characterize it, sometimes we abuse the notation writing e.g.: $C \setminus E(D)$, with *D* a subgraph of *G*, to denote the chain graph induced by edges $E(C)\setminus E(D)$; $C \subseteq E(D)$ or equivalently $C \subseteq D$ to say that *C* is an edge-induced subgraph of *D* and $e \in C$ to mean that $e \in E(C)$.

A *maximal chain subgraphC* of a given bipartite graph *G* is a connected chain subgraph such that no superset of *E*(*C*) is a chain subgraph. We denote by ${\mathscr {C}}(G)$ the set of all maximal chain subgraphs in *G*.

A set of chain subgraphs $C_1, \ldots , C_k$ is a *cover* for *G* if $\cup _{1\le i \le k} E(C_i)=E(G)$. Observe that, given any cover of *G* by chain subgraphs $C=\{ C_1, \ldots C_k \}$, there exists another cover of same size $C'=\{ C'_1, \ldots C'_k \}$ whose chain subgraphs are all maximal; more precisely, for each $i=1, \ldots , k$, $C'_i$ is a maximal chain subgraph of *G* and $C'_i$ admits $C_i$ as subgraph. In order to avoid redundancies, from now on, although not explicitly highlighted, we will restrict our attention to the covers by maximal chain subgraphs.

We denote by ${\mathcal {S}}(G)$ the set of all minimal chain covers of a bipartite graph *G*.

## Modeling cytoplasmic incompatibility

In [6, 9], the cytoplasmic compatibility relationships that are observed in a given dataset are represented as a binary $n_1\times n_2$ matrix *C* with the males in rows and females in columns. Entry $C[i,j]= 0$ represents a compatible cross (offspring survival) between male *i* and female *j*; $C[i,j]= 1$ representing an incompatible cross. Under the quantitative Lock/Key model (see Section Quantitative Model in [9]) the unknown infections with *Wolbachia* strains are represented as an $n_1 \times k$ matrix *L* and an $n_2 \times k$ matrix *K* that describe the Lock and Key factors carried by the host males and females, respectively. Matrices *L* and *K* contain integer values and for each entry $L[i,l]=q$ ($K[j,t]=q$), the value *q* indicates that the Lock molecule *l* is found in quantity *q* in male *i* (the Key molecule *t* is found in quantity *q* in female *j*). A value of *q* equal to 0 indicates the absence of the molecule in the host. The pattern observed in *C* can be explained by the matrices *K* and *L* in the following way: $C[i,j]=0$, i.e. the crossing between male *i* and female *j* is successful, if and only if female *j* has *enough* Key molecules to ”open” all the Lock molecules. More formally,

### **Definition 1**

Given an incompatibility $n_1\times n_2$ matrix *C*, an $n_1 \times k$ matrix *L* and an $n_2 \times k$ matrix *K*, we say that *L*, *KexplainC* if the following holds: $C[i,j]=0$ if and only if for all $1\le l \le k$ it holds that $K[j,l] \ge L[i,l]$.

In a parsimonious context, the goal is, given a matrix *C*, to find two matrices *L*, *K* that explain all the crosses in *C* and have a minimum number of columns *k*.

This problem has been formulated in [24] in terms of graphs. Matrix *C* can be seen as the adjacency matrix of a bipartite graph $B(C)=(U \cup W, E)$, with males in *U* and females in *W* and edges representing the incompatible crosses. We include this formulation here for the completeness of the paper.

### **Lemma 1**

*Given an incompatibility*$n_1 \times n_2$*matrixC*, *the bipartite graph*$B(C)=(U \cup W, E)$*is a chain graph if and only if there exist an*$n_2 \times 1$*matrixLand*$n_1 \times 1$*matrixKthatexplainC*.

### *Proof*

We start by first showing the reduction between those two representations. Let *C* be an incompatibility $n_1 \times n_2$ matrix, we first assume that $B(C)=(U\cup W, E)$ is a chain graph. Let $U=\{u_1, \ldots , u_{n_1}\}$ and $W=\{w_1,\ldots , w_{n_2}\}$. By definition of a chain graph, we can assume that the nodes of *U* can be linearly ordered such that $N_G(u_1) \subseteq N_G(u_2)\subseteq \ldots \subseteq N_G(u_{n_1})$. Note that for all $i<j$ it holds $|N_G(u_i)| \le |N_G(u_j)|$, hence it is possible to group the nodes of *U* in *d* classes $B_1, \ldots , B_d$ such that a node $u_i \in U$ belongs to $B_r$ (with $r \le d$) if and only if $|N_G(u_i)|=r$. Note that some of the $B_i$ can be empty and in $B_0$ we have all the isolated nodes (if any) of *U*. If $u_i,u_j \in B_r$ then $N(u_i)=N(u_r)$, hence we will extend the notion of neighbourhood and denote it by $N(B_r)=N(u)$ for some $u \in B_r$.

We show that one pair Lock/Key is sufficient to explain the matrix *C* observed. To this purpose we construct the matrices *L* and *K* that explain *C* as follows: for all $u_i \in B_j$ we assign $L[i][1]=j$, and for all $w_t \in N(B_j) \setminus \cap _{l=1}^ {j-1}N(B_l)$ we assign $K[t][1]=j-1$.

Intuitively, we assign to a node $u \in U$ a quantity of the Lock molecule that depends on its degree. Also all the nodes of *W* in the neighbourhood of *u* should have a smaller quantity such that the cross results incompatible. Notice that the same reduction works in the opposite direction.

The lemma follows by the chain of equivalences: $$C[i][j]=1$$$$\Leftrightarrow$$$$(u_i, w_j)$$ in *B*(*C*) $$\Leftrightarrow$$$$u_i \in B_s$$ for some $$1\le s \le d$$ and $$w_j \in \cap _{l=1}^ {s}N(B_l)$$$$\Leftrightarrow$$ by construction $$L[i][1]=i$$ and $$K[j][1]<i$$. $$\square$$

**Fig. 2:**
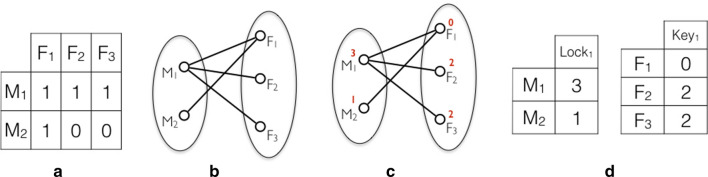
Example of the quantitative model for CI. **a** An example of an incompatibility matrix. **b** The corresponding bipartite graph. **c** The output of the quantitative model. **d** The lock and key matrices with the corresponding quantities for the males and females. This dataset can be explained by only one strain of Wolbachia and the quantities of the molecules are such that a cross is successful if and only if quantity of the Key molecule in the female is at least as the one of the Lock molecule in the male

An example that illustrates the connection between incompatibility matrix, bipartite graph and chain graph is depicted in Fig. 2. Given the incompatibility matrix in Fig. 2a, the corresponding bipartite graph is constructed in Fig. 2b. Recall that an edge corresponds to a cross incompatibility. We apply the procedure described in the proof of Lemma 1 and thus we can define $B_3=\{M_1\}, B_2=\emptyset , B_1=\{M_2\}$, $F_1 \in N(B_1)$ and $F_2 , F_3 \in N(B_3) \setminus \cap _{l=1}^ {2}N(B_l)$. We can explain the dataset with only one pair of Lock-Key molecule. The assignment of the quantities of the lock and key molecules to the males and females is done as described before and is depicted in Fig. 2d. It is not difficult to check that the cross between male *i* and female *j* is successful if and only if the female has enough of the key molecules for the lock molecules of the male.

The straightforward outcome of the lemma is that a matrix representation of any chain subgraph *B* can be represented by exactly one pair of Lock/Key molecules. Hence, the next theorem follows.

### **Theorem 1**

*Given an incompatibility*$n_1 \times n_2$*matrixC*, *there exist an*$n_1 \times k$*matrixLand an*$n_2 \times k$*matrixKthatexplainCif and only if the bipartite graphB*(*C*) *has an edge cover withkchain subgraphs.*

The previous theorem motivates the problems we study in this paper.

## Enumerating all maximal chain subgraphs

In this section, we provide a polynomial delay algorithm for enumerating all the maximal chain subgraphs of a given bipartite graph. We start by proving the following result.

### **Proposition 1**

*Let*$C=(X \cup Y, F)$*be a chain subgraph of*$G=(U \cup W, E)$, *with*$X \subseteq U$, $Y \subseteq W$*and*$F \subseteq E$, *and let*$x \in X$*be a node with largest neighbourhood inC*. *ThenCis a maximal chain subgraph ofGif and only if both the following conditions hold:*

(i) $N_C(x) = N_G(x)$*is a maximal neighbourhood ofG*, *i.e.there does not exist a node*$x' \in X$*such that*$N_G(x) \subset N_G(x')$;
(ii) $C \setminus E_G(x)$*is a maximal chain subgraph of*$G\big [U \setminus \{x\}, N_G(x)\big ]$.

### *Proof*

($\Rightarrow$) Let $C=(X\cup Y, F)$ be a maximal chain subgraph of $G=(U\cup W, E)$. To prove that (i) holds, suppose by contradiction that $N_C(x)$ is not a maximal neighbourhood of *G*, i.e. there exists $x' \in U$ with $N_C(x) \subset N_G(x')$ (possibly $x' = x$). Since $N_C(x)$ is the largest neighbourhood of *C*, for all $z\in X$, we have $N_C(z) \subseteq N_C(x) \subset N_G(x')$, so we can add to *C* all the edges incident to $x'$ and still obtain a chain subgraph thereby contradicting the maximality of *C*.

To prove that *(ii)* holds, first observe that $N_G(x)=Y$ (otherwise we would violate (i) with $x'=x$). By contradiction, assume that $C \setminus E_G(x)$ is not maximal in $G\big [U \setminus \{x\}, N_G(x)\big ]$. Then, there exists a chain subgraph $C'$ such that $C \setminus E_G(x) \subset C' \subseteq G\big [U \setminus \{x\}, N_G(x)\big ]$. By adding to each one of the previous graphs the edges in $E_G(x)$, we have that the strict inclusion is preserved because the added edges were not present in any one of the three graphs. Since $C'$ with the addition of $E_G(x)$ is still a chain subgraph with $N_G(x)$ as its largest neighbourhood, we reach a contradiction with the hypothesis that *C* is maximal in *G*.

($\Leftarrow$) We show that if both (i) and (ii) hold, then the chain subgraph *C* of *G* is maximal. Suppose by contradiction that *C* is not maximal in *G*, and let $C'$ be a chain subgraph of *G* such that $C \subset C'$. Let *x* be the node with the largest neighbourhood in *C*. It follows that $N_{C}(x)\subseteq N_{C'}(x)$. As (i) holds, we have that $N_G(x) = N_C(x) \subseteq N_{C'}(x) \subseteq N_G(x)$ from which we derive that $N_{C'}(x) = N_G(x)$, and that $C' \subseteq G\big [U, N_G(x)\big ]$ since $N_{C'}(x)$ is a maximal neighbourhood of *G*, hence the largest neighbourhood of $C'$ (and *C* by the hypothesis). This implies also that *C* and $C'$ differ in some node different from *x*, i.e. $C \setminus E_G(x) \subset C' \setminus E_G(x) \subseteq G\big [U \setminus \{x\}, N_G(x)\big ]$. Notice that $C' \setminus E_G(x)$ is still a chain subgraph because we simply removed node *x* and all its incident edges. We then get a contradiction with *(ii)*. $\square$

Proposition 1 leads us to design Algorithm 1 which efficiently enumerates all maximal chain subgraphs of *G*. It exploits the fact that, in each maximal chain subgraph, a node *u* whose neighbourhood is largest is also maximal in *G* (part (i) of Proposition 1) and this holds recursively in the chain subgraph obtained by removing node *u* and restricting the graph to $N_C(u)$ (part (ii) of Proposition 1). To compute the maximal neighbourhood nodes, the algorithm uses function computeCandidates that, given sets *U* and *W*, for each maximal neighbourhood $Y\subset W$, returns a unique node *u*, called *candidate*, for which $N_G(u)=Y$. This means that in case of twins, function computeCandidates extracts only one representative node according to some fixed order on the nodes (e.g. the node with the smallest label). If the graph has no edges, the function returns the empty set. 
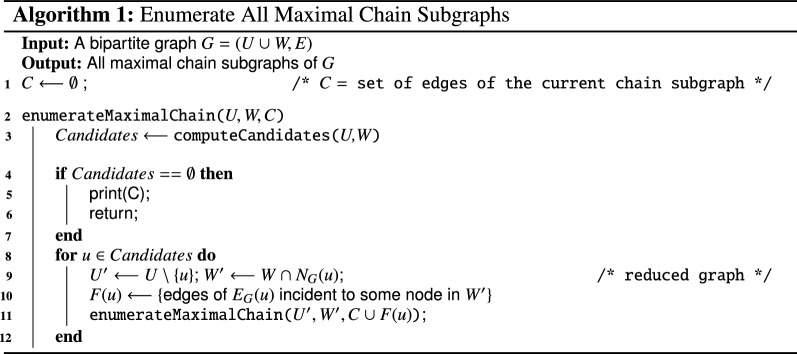


### **Proposition 2**

(Correctness) *Algorithm 1 correctly enumerates all the maximal chain subgraphs of the input graphGwithout repetitions.*

### *Proof*

Let $G=(U \cup W, E)$ be a bipartite graph. We prove the correctness of Algorithm 1 by induction on |*U*|, i.e. we show that all the solutions are output, without repetitions.

When $|U| = 1$, let *u* be the only node in *U*. We have that $N_G(u)$ is the only neighbourhood in *W*, and line 3 returns $\{u\}$ as unique candidate. In line 9, the algorithm reduces the graph of interest. In line 10, the whole $E_G(u)$ is added to the current chain subgraph *C*. Then the function is recursively recalled, with $U' = \emptyset$ so the condition at line 4 is true and *C* is printed; it is in fact the only chain subgraph of *G*, it is trivially maximal and there are no repetitions. Correctness then follows when $|U| = 1$.

Assume now that $|U|=k$ with $k>1$. As inductive hypothesis, let the algorithm work correctly when $|U| \le k-1$.

For each candidate *u*, the algorithm recursively recalls the same function on a reduced subgraph and, by the inductive hypothesis, outputs all chain subgraphs of this reduced subgraph without repetitions. By Proposition 1, if we add to each one of these chain subgraphs the node *u* and all the edges incident to *u* in *G*[*U*, *W*], we get a different maximal chain subgraph of *G* since each maximal chain subgraph has one and only one maximal neighborhood and the function computeCandidates returns only one representative node. Recall that in the case of twin nodes the algorithm will always consider the nodes in a precise order and so no repetition occurs. Moreover, iterating this process for all candidates guarantees that all maximal chain subgraphs are enumerated and no one is missed. $\square$

Let $G=(U \cup W, E)$ be a bipartite graph, with $n=|U|+|W|$ and $m = |E|$. Before proving the time complexity of Algorithm 1, we observe that the running time of function ComputeCandidates is *O*(*nm*). Indeed, for each node $u_i \in U$, it requires only time proportional to $\sum _{j=1}^{i-1} \big (deg(u_j) + deg(u_i) \big )$ to check whether the neighbourhood of $u_i$ either is included, or includes the neighbourhood of $u_j$, for each $j<i$, assuming that the adjacency lists of the graph are ordered. So, the time complexity is upper bounded as follows:

$$\begin{aligned} & \sum _{i=1}^n \sum _{j=1}^{i-1}(deg(u_j)+deg(u_i)) \le \sum _{i=1}^n (\Theta (m)+(i-1) deg(u_i)) \hfill \\ & \quad \quad \quad \le \Theta (nm)+n \sum _{i=1}^n deg(u_i)=\Theta (nm). \end{aligned}$$

### **Proposition 3**

(Time Complexity and Polynomial Delay) *Let*$G=(U \cup W, E)$*be a bipartite graph. The total running time of Algorithm 1 is*$O(|{\mathscr {C}}(G)| n^2 m)$*where*$|{\mathscr {C}}(G)|$*is the number of maximal chains subgraph ofG*. *Moreover, the solutions are enumerated in polynomial time delay*$O(n^2 m)$.

### *Proof*

Represent the computation of Algorithm 1 as a tree of the recursion calls of enumerateMaximalChain, each node of which stores the current graph on which the recursion is called at line 11. Of course, the root stores *G* and on each leaf the condition $Candidates == \emptyset$ is true and a new solution is output. Observe that each leaf contains a feasible solution, and that no repetitions occur in view of Proposition 2, so the number of leaves is exactly $|{\mathscr {C}}(G)|$.

Since at each call the size of *U* is reduced by one, the tree height is necessarily bounded by $|U|=O(n)$; moreover, on each tree node, *O*(*nm*) time is spent for running function ComputeCandidates.

It follows that, since the algorithm explores the tree in DFS fashion starting from the root, between two solutions the running time is at most $O(n^2 m)$ and the total running time is $O(|{\mathscr {C}}(G)| n^2 m)$. $\square$

## Upper bounds on the number of maximal chain subgraphs

In this section, we give two upper bounds on the maximum number of maximal chain subgraphs of a bipartite graph *G* with *n* nodes and *m* edges. The first bound is given in terms of *n* while the second depends on *m*. These bounds are of independent interest, however we will use them in two directions. First, they will allow us to determine a (input-sensitive) time complexity of Algorithm 1. Indeed, in Proposition 3, we proved that the total running time of Algorithm 1 is of the form $O(D(n) \cdot |{\mathscr {C}}(G)|)$, where *D*(*n*) is the delay of the algorithm and $|{\mathscr {C}}(G)|$ is the number of maximal chain subgraphs of *G*. Thus, a bound on $|{\mathscr {C}}(G)|$ leads to a bound on the running time of Algorithm 1 depending on the size of the input. Second, the bound on $|{\mathscr {C}}(G)|$ in terms of edges allows us to compute the time complexity of an exact exponential algorithm for the minimum chain subgraph cover problem in “Minimum chain subgraph cover” section.

### Bound in terms of nodes

The following lemma claims that a given permutation is the neighbourhood ordering of at most one maximal chain subgraph.

#### **Lemma 2**

*Let*$C_1$*and*$C_2$*be two maximal chain subgraphs of*$G=(U\cup W,E)$*and let*$\pi _{1}$ (*resp.*$\pi _{2}$) *be a neighbourhood ordering of*$C_1$ (*resp.*$C_2$). *Then*, $\pi _{1}=\pi _{2} \Longrightarrow C_1=C_2$.

#### *Proof*

The proof proceeds by induction on the number of nodes of *U*.

If $|U|=1$ then *G* has only one maximal chain subgraph and the result trivially holds.

Assume now that $|U|>1$. By Proposition 1, we have that $N_{C_1}(u_{\pi (|U|)})=N_{G}(u_{\pi (|U|)})=N_{C_2}(u_{\pi (|U|)})$. Using again Proposition 1, we obtain that $C'_{1}:=C_1[U\setminus \{u_{\pi (|U|)}\}, N_{G}(u_{\pi (|U|)})]$ and $C'_{2}:=C_2[U\setminus \{u_{\pi (|U|)}\} , N_{G}(u_{\pi (|U|)})]$ are maximal chain subgraphs of the graph defined as $G[U\setminus \{u_{\pi (|U|)}\}, N_{G}(u_{\pi (|U|)})]$. Applying the inductive hypothesis with the permutations restricted to the $|U|-1$ elements, we have that $C'_{1}=C'_{2}$. Finally, since $N_{C_1}(u_{\pi (|U|)})=N_{C_2}(u_{\pi (|U|)})$, we conclude that $C_1=C_2$. $\square$

As a corollary, the maximum number of chain subgraphs of a graph $G=(U\cup W,E)$ is bounded by |*U*|!. Since the same reasoning can be applied on *W*, we have that $|{\mathscr {C}}(G)|\le |W|!$ and hence:

$$\begin{aligned} |{\mathscr {C}}(G)|\le \min (|U|,|W|)!\le \frac{n}{2}! \end{aligned}$$

This bound is tight as shown by the following family of graphs that reaches it.

Consider the *antimatching graph with n nodes*$A_{n} =( U \cup W, E)$ defined as the complement of an *n*/2 edge perfect matching, i.e.:

$$\begin{aligned} U&{:=}\{u_1,\dots , u_{n/2}\}, \quad W{:}= \{w_1,\dots , w_{n/2}\}, \\ E&{:=}\{(u_i,w_j) \in U \times W{:} i \ne j \}. \end{aligned}$$

It is not difficult to convince oneself that the maximal chain subgraphs of $A_{n}$ are exactly (*n*/2)! and that a different permutation corresponds to each of them. In particular, for each permutation $\pi$ of the nodes of *U*, the corresponding maximal chain subgraph $C_{\pi }$ of $A_{n}$ can be defined by means of the set of neighbourhoods as follows:

$$\begin{aligned} N_{C_{\pi }}(u_i){:=}\{ w_k\text { s.t. }\pi ^{-1}(k)<\pi ^{-1}(i)\}. \end{aligned}$$

$C_{\pi }$ is a chain subgraph since all the neighbourhoods form a chain of inclusions. Moreover, it is maximal since if we added to the neighbourhood of $u_{i}$ any one of the missing edges $(u_{i},w_{j})$ with $\pi ^{-1}(j) \ge \pi ^{-1}(i)$, we would introduce a $2K_2$ with the existing edge $(u_{j},w_{i})$ as $(u_{j}, w_{j})$ and $(u_{i},w_{i})$ are not in *E*.

### Bound in terms of edges

Let *T*(*m*) be the maximum number of maximal chain subgraphs over all bipartite graphs with *m* edges. After two preliminary lemmas, we prove that $T(m)\le 2^{\sqrt{m}\log (m)}$.

#### **Lemma 3**

*Let*$G=(U \cup W, E)$*be a bipartite graph. Then*$|{\mathscr {C}}(G)| \le |U| \cdot T(m-|W|)$.

#### *Proof*

In view of how the algorithm works and of Proposition 1, at the beginning, there at most |*U*| candidates. For each candidate *x*, we can build as many chain subgraphs as there are in $G[U \setminus \{ x \}, N_G(x)]$. We claim that this latter graph has at most $m-|W|$ edges. Indeed, in order to construct $G[U \setminus \{ x \}, N_G(x)]$, we remove from *G* exactly $|E_G(x)|$ edges when deleting *x* from *U*, and $|W| - |N_G(x)|$ nodes (each one connected to at least a different edge as *G* is connected) when reducing *W* to $N_G(x)$. Observing that $|E_G(x)|=|N_G(x)|$, in total we remove at least |*W*| edges. It is not difficult to see that *T*(*m*) is increasing with *m*. Hence, the proof follows from the fact that the number of chain subgraphs of $G[U \setminus \{ x \}, N_G(x)]$ is bounded by $T(m-|W|)$. $\square$

By the next Lemma, we have that the maximum on *n* of the auxiliary function $\frac{n}{2}\cdot 2^{\sqrt{m-\frac{n}{2}} \log (m-\frac{n}{2})}$ is reached when *n*/2 is minimum (note that trivially for a bipartite graph we have $n/2 > \sqrt{m}$).

#### **Lemma 4**

*The real-valued function*$F(x):= x2^{\sqrt{m-x} \log (m-x)}$*is decreasing in the interval*$[\sqrt{m},m-1]$.

#### *Proof*

The derivative of *F*(*x*) is given by:

$$\begin{aligned}&-x {\left( \frac{\log \left( m - x\right) }{2\sqrt{m - x}} + \frac{1}{\sqrt{m - x}}\right) } 2^{\left( \sqrt{m - x} \log \left( m - x\right) \right) } + 2^{\left( \sqrt{m - x} \log \left( m - x\right) \right) }\\&\quad = -\frac{{\left( x \log \left( m - x\right) + 2x - 2\sqrt{m - x}\right) } 2^{\left( \sqrt{m - x} \log \left( m - x\right) \right) }}{2\sqrt{m - x}} \end{aligned}$$

Then the derivative is negative whenever $\left( x \log \left( m - x\right) + 2x - 2\sqrt{m - x}\right) \ge 0$.

Observe that $\log \left( m - x\right) \ge 0$ for $x \le m - 1$, while for $x \ge 0$ we have:

$$\begin{aligned} 2x - 2\sqrt{m - x} \ge 0 \Longleftrightarrow x \ge \frac{-1 + \sqrt{ 1 + 4m}}{2} = -\frac{1}{2} + \sqrt{m + \frac{1}{4}} \end{aligned}$$

and:

$$\begin{aligned} \sqrt{m} \ge -\frac{1}{2} + \sqrt{m + \frac{1}{4}} \end{aligned}$$

$$\square$$

We are now able to prove the main theorem of this subsection:

#### **Theorem 2**

*Let*$G=(U \cup W,E)$*be a bipartite graph withnnodes andmedges; then*$|{\mathscr {C}}(G)|\le 2^{\sqrt{m} \log m}$, i.e. $T(m)\le 2^{\sqrt{m} \log m}$.

#### *Proof*

Assume w.l.o.g that $|U| \le |W|$. The proof is by induction on *m*. Note that for $m=1$ the theorem trivially holds. Applying the inductive hypothesis and Lemma 3, we have:

$$\begin{aligned} |{\mathscr {C}}(G)|\le |U|T(m-|W|) \le \frac{n}{2} 2^{\left( \sqrt{m - \frac{1}{2} \, n} \log \left( m - \frac{1}{2} \, n\right) \right) }. \end{aligned}$$

Since the function $x \mapsto x2^{\sqrt{m-x} \log (m-x)}$ is decreasing in the interval $[\sqrt{m},m-1]$, the maximum of $\frac{n}{2} 2^{\sqrt{m-\frac{n}{2}} \log (m-\frac{n}{2})}$ is reached when *n*/2 is minimum. Note that trivially for a bipartite graph we have $n/2 > \sqrt{m}$. Hence,

$$\begin{aligned} |{\mathscr {C}}(G)|\le \sqrt{m}\, 2^{\sqrt{m-\sqrt{m}} \log (m-\sqrt{m})} \end{aligned}$$

Let $A:= \sqrt{m}-\sqrt{m-\sqrt{m}}$ and $B:= \frac{m-\sqrt{m}}{m}$. Then:

$$\begin{aligned} \begin{array}{lll} |{\mathscr {C}}(G)|&{}\le &{} \sqrt{m}\, 2^{(\sqrt{m}-A) \log (mB)}\\ &{}=&{} 2^{\sqrt{m}\log m} \times \sqrt{m}\,2^{ \log {B}(\sqrt{m} - A) \; - \; A\log m} \end{array} \end{aligned}$$

Let us show that $Z:=\sqrt{m}\,2^{ (\sqrt{m} - A)\log B\;-\;A\log m} \le 1$ by showing that $\log {Z}\le 0$:

$$\begin{aligned}\begin{array}{lll} \log {Z}&{}= &{} \log (\sqrt{m}) + (\sqrt{m} -A)\log B \; - \; A\log m \\ &{}=&{} (1 -2A)\log (\sqrt{m}) + (\sqrt{m} - A)\log B\\ &{}\le &{} 0 \end{array}\end{aligned}$$

considering that $B < 1$ and $1/2<A \le 1$ since:

$$\begin{aligned} A = \frac{1}{1+\sqrt{B}} = \frac{1}{1 + \sqrt{ 1 - \frac{1}{\sqrt{m}}}} \end{aligned}$$

$$\square$$

By this bound on the number of maximal chain subgraphs we trivially obtain an input-sensitive bound on the time complexity for Algorithm 1:

#### **Corollary 1**

*The (input-sensitive) complexity of Algorithm 1 is bounded by*$O^*(2^{\sqrt{m} log(m)})$.

## Minimum chain subgraph cover

In this section, we show how to find in polynomial space the minimum size of a chain subgraph cover in time $O^*((2+\epsilon )^{m})$, for every $\varepsilon >0$. Since a chain subgraph cover is a family of subsets of edges, the existence of an algorithm whose complexity is close to $2^{m}$ is not obvious. Indeed the basic search space has size $2^{2^{m}}$, as it corresponds to a family of subsets of edges. To obtain this result, we exploit Algorithm 1, the bound obtained in Theorem 2 and the inclusion/exclusion method [16, 22] that has already been successfully applied to exact exponential algorithms for many partitioning and covering problems.

We first express the problem as an inclusion-exclusion formula over the subsets of edges of *G*.

### **Proposition 4**

[22] *Let*$c_k(G)$*be the number of chain subgraph covers of sizekof a graphG*. *We have that:*

$$\begin{aligned} c_{k}(G)= \sum _{A\subseteq E}(-1)^{|A|}a(A)^{k} \end{aligned}$$

*wherea*(*A*) *denotes the number of maximal chain subgraphs not intersectingA*.

Exploiting this result, we can design an exact algorithm which counts the number of chain subgraph covers of size *k* with a time complexity given in the following theorem:

### **Theorem 3**

*Given a bipartite graphGwithmedges, for all*$k\in \mathbb {N}^{*}$*and for all*$\varepsilon >0$, *the number of chain subgraph covers of sizek*, *denoted with*$c_{k}(G)$, *can be computed in time*$O^*((2+\epsilon )^{m})$.

### *Proof*

Let $G=(U \cup W,E)$ be a bipartite graph, $k \in \mathbb {N}^{*}$ and fix an $\varepsilon >0$. Using the formula of Proposition 4, $c_{k}$ can be computed in time $$\sum \limits _{i=0}^{m}\left( {\begin{array}{c}m\\ i\end{array}}\right) C(i)$$, where *C*(*i*) is the time complexity needed to compute *a*(*A*), $|A|=i$.

Notice that to compute *a*(*A*) for a given $A \subseteq E$, one can naively compute all maximal chain subgraphs of $G'=(U \cup W,E\setminus A)$ and, for each of them, check whether it is maximal in *G*. Using this fact and Corollary 1, *C*(*i*) can be determined in time $O(n^2 m 2^{\sqrt{m-i} \log (m-i)})$.

Thus we have that $c_k(G)$ can be computed in time $$\sum \limits _{i=0}^{m}\left( {\begin{array}{c}m\\ i\end{array}}\right) n^2 m 2^{\sqrt{m-i} \log (m-i)}$$. Observe now that since $2^{\sqrt{m-i} \log (m-i)}=o((1+\varepsilon )^m)$, there exists a constant $n_{\varepsilon }$ such that for all $m>n_{\varepsilon }$, $2^{\sqrt{m-i} \log (m-i)}< (1+\varepsilon )^m$. We have that:

$$\begin{aligned} \sum \limits _{i=0}^{m}\left( {\begin{array}{c}m\\ i\end{array}}\right) n^2 m 2^{\sqrt{m-i} \log (m-i)} & = {} n^2 m \Biggl (\sum \limits _{i=0}^{m-n_{\varepsilon } -1}\left( {\begin{array}{c}m\\ i\end{array}}\right) 2^{\sqrt{m-i} \log (m-i)} + \sum \limits _{i=m-n_{\varepsilon }}^{ m}\left( {\begin{array}{c}m\\ i\end{array}}\right) 2^{\sqrt{m-i} \log (m-i)} \Biggr ) \\ & \quad \le {} n^2 m \Biggl ( \sum \limits _{i=0}^{m-n_{\varepsilon } -1}\left( {\begin{array}{c}m\\ i\end{array}}\right) (1+\varepsilon )^{m-i}+ n_{\varepsilon } m^{n_{\varepsilon }} 2^{\sqrt{n_{\varepsilon }} \log (n_{\varepsilon })} \Biggr ) \\ & \quad \le {} n^2 m \Biggl ( \sum \limits _{i=0}^{m}\left( {\begin{array}{c}m\\ i\end{array}}\right) (1+\varepsilon )^{m-i} + n_{\varepsilon } m^{n_{\varepsilon }} 2^{\sqrt{n_{\varepsilon }} \log (n_{\varepsilon })} \Biggr ) \\ & \quad \le {} n^2 m (2+\varepsilon )^{m} + n^2 n_{\varepsilon } m^{1+n_{\varepsilon }} 2^{\sqrt{n_{\varepsilon }} \log (n_{\varepsilon })}\\ & ={} O^*((2+\varepsilon )^{m}). \end{aligned}$$

Where the last step follows by recalling that *G* is connected and thus $n = O(m)$. $\square$

We conclude, by observing that the size of a minimum chain cover is given by the smallest value of *k* for which $c_{k}(G)\ne 0$.

## Enumeration of minimal chain subgraph covers

In this section, we prove that the enumeration of all minimal chain subgraph covers can be polynomially reduced to the enumeration of the minimal set covers of a hypergraph. This reduction implies that there is a quasi-polynomial time algorithm to enumerate all minimal chain subgraph covers. Indeed, the result in [25] implies that all the minimal set covers of a hypergraph can be enumerated in time $N^{\log {N}}$ where *N* is the sum of the input size (i.e. $n+m$) and of the output size (i.e. the number of minimal set covers).

Let them $G=(U \cup W,E)$ be a bipartite graph, ${\mathscr {C}}={\mathscr {C}}(G)$ the set of all maximal chain subgraphs of *G* and ${\mathcal {S}}={\mathcal {S}}(G)$ the set of minimal chain subgraph covers of *G*. Notice that the minimal chain subgraph covers of *G* are the minimal set covers of the hypergraph ${\mathcal {H}}=(V,{\mathcal {E}})$ where $V=E$ and ${\mathcal {E}}={\mathscr {C}}$. Unfortunately, the size of ${\mathcal {H}}$ might be exponential in the size of *G* plus the size of ${\mathcal {S}}$. Indeed not every maximal chain subgraph in ${\mathscr {C}}$ will necessarily be part of some minimal chain subgraph cover. To obtain a quasi-polynomial time algorithm to enumerate all minimal chain subgraph covers, we need to enumerate only those maximal chain subgraphs that belong to a minimal chain subgraph cover.

Given an edge $e \in E$, let ${\mathscr {C}}_e$ be the set of all maximal chain subgraphs of *G* containing *e*.

We call an edge $e \in E$*non-essential* if there exists another edge $e' \in E$ such that ${\mathscr {C}}_{e'} \subset {\mathscr {C}}_{e}$. An edge which is not non-essential is said to be *essential*. Note that for every non-essential edge *e*, there exists an essential edge $e_1$ such that ${\mathscr {C}}_{e_1} \subset {\mathscr {C}}_{e}$. Indeed, by applying iteratively the definition of a non-essential edge, we obtain a list of inclusions ${\mathscr {C}}_{e}\supset {\mathscr {C}}_{e_1} \supset {\mathscr {C}}_{e_2} \ldots$, where no ${\mathscr {C}}_{e_i}$ is repeated as the inclusions are strict. The last element of the list will correspond to an essential edge.

The following lemma claims that if a maximal chain subgraph *C* contains at least one essential edge, then it belongs to at least one minimal chain subgraph cover.

### **Lemma 5**

*LetCbe a maximal chain subgraph of a bipartite graph*$G=(U \cup W,E)$. *ThenCbelongs to a minimal chain subgraph cover ofGif and only ifCcontains an essential edge.*

### *Proof*

($\Rightarrow$) Let *C* belong to a minimal chain subgraph cover *M* and assume that *C* contains no essential edge. Given $e \in C$, *e* therefore being non-essential, there exists an essential edge $e'$ such that ${\mathscr {C}}_{e'} \subset {\mathscr {C}}_{e}$. Moreover, $e' \not \in C$. As *M* is a cover, there exists $C' \in M$ such that $e' \in C'$. Thus, $C' \ne C$, $C' \in {\mathscr {C}}_{e'} \subset {\mathscr {C}}_{e}$, hence $e \in C'$. Since for every edge $e \in C$, there exists $C' \in M$ containing it, we have that $M\setminus \{C\}$ is a cover, contradicting the minimality of *M*.

($\Leftarrow$) Assume *C* contains an essential edge *e*. Let ${\mathscr {C}}'=\{D \in {\mathscr {C}}(G): e \not \in D\}$. Note that ${\mathscr {C}}' = {\mathscr {C}}\setminus {\mathscr {C}}_{e}$. We show that ${\mathscr {C}}' \cup \{C\}$ is a cover. Suppose on the contrary that there exists $e' \in E \setminus E(C)$ and $e'$ is not covered by ${\mathscr {C}}'$ and thus ${\mathscr {C}}_{e'} \cap {\mathscr {C}}'=\emptyset$. This implies that ${\mathscr {C}}_{e'} \subseteq {\mathscr {C}}\setminus {\mathscr {C}}'={\mathscr {C}}_{e}$ and as *e* is essential, we obtain ${\mathscr {C}}_{e'}= {\mathscr {C}}_{e}$ from which we deduce that $e' \in C$. Thus, $M={\mathscr {C}}' \cup \{C\}$ is a cover and clearly it contains a minimal one. Finally, we conclude by observing that, since by construction *C* is the only chain subgraph of *M* that contains *e*, it belongs to any minimal cover contained in *M*. $\square$

It follows that the set of maximal chain subgraphs that can contribute to a minimal chain cover is $\tilde{{\mathscr {C}}}= \cup {\mathscr {C}}_e$ where the index *e* runs over all the essential edges of *G*. In the following, we show how to detect essential edges. This problem then consists in detecting all the couples $e_1$, $e_2$ such that ${\mathscr {C}}_{e_1} \subseteq {\mathscr {C}}_{e_2}$ before enumerating all useful maximal chain subgraphs.

Theorem 4 later in this section provides an efficient way to detect these couples. In order to prove it, we need first some preliminary results.

Let ${\mathcal {M}}_e$ the set of all edges $e' \in E$ inducing a $2K_2$ in *G* together with *e*.

### Fact 1

*Let*$C=(X\cup Y, F)$*be a maximal chain subgraph of a bipartite graph*$G=(U\cup W, E)$, *and let*$z\in X$, $e=\{u,w\} \in E$*be such that for every*$e' \in E_C(z)$, *we have*$e\not \in {\mathcal {M}}_{e'}$. *Then at least one of the following holds:*

- $$w\in N_G(z)$$.
- $$u \in \cap _{y\in N_C(z)} N_G(y)$$.

### *Proof*

The proof follows straightforwardly by observing that for any $e'=\{z,y\}\in C$ then as $e\not \in {\mathcal {M}}_{e'}$, either $\{z,w\}\in E(G)$ or $\{u,y\} \in E(G)$. $\square$

Observe that in the previous claim, we can re-write (b) in the form:

(b’) $$N_C(z) \subseteq N_G(u)$$.

### **Lemma 6**

*LetCbe a maximal chain subgraph of a bipartite graph*$G=(U \cup W, E)$*and let*$e \in E$*be such that for all*$e' \in E(C)$, *it holds that*$e \not \in {\mathcal {M}}_{e'}$. *Then*$e\in C$.

### *Proof*

Let $C=(X\cup Y, F)$ be a maximal chain subgraph of $G=(U \cup W, E)$ and w.l.o.g., let $u_1,\dots , u_{|X|} \in X \subseteq U$ such that $N_C(u_1)\subseteq N_C(u_2)\subseteq \ldots \subseteq N_C(u_{|X|})$. We can furthermore assume that *C* is connected.

From the hypothesis, let $e=\{u,w\}$ in *E* be such that for all $e' \in E(C)$, it holds $e \not \in {\mathcal {M}}_{e'}$. Finally, assume $e \not \in C$.

The proof runs by contradiction: we will show that

1 $$\begin{aligned} w \in \cap _{x\in X}N_G(x) \end{aligned}$$

must hold. Although, this contradicts the maximality of *C* as in this way we could add *e* and all the other edges in $E_G(w)$ to *C* and still obtain a chain subgraph (with $N_G(w)$ as the largest neighbourhood of *C*).

In order to prove (1), using Fact 1 with $z=u_{|X|}$, we have that at least one among (a) and (b) must hold. Observe that (b) cannot hold as otherwise we have straightaway (1) (interchanging the roles of *Y* and *X*, and *w* and *u*) observing that $N_C(u_{|X|}) = N_G(u_{|X|}) =Y$ by point (i) of Proposition 1. Thus, (a) must hold, i.e. $w\in N_G(u_{|X|})$.

If we now show that $w\in \cap _{k=j}^{|X|}N_G(u_k) \Rightarrow w\in N_G(u_{j-1})$, we prove the claim since together with the just proved $w\in N_G(u_{|X|})$ this leads to (1):

$$\begin{aligned} w\in \bigcap _{k=1}^{|X|}N_G(u_k) = \bigcap _{x\in X}N_G(x) \end{aligned}$$

We conclude the proof by showing the validity of $w\in \cap _{k=j}^{|X|}N_G(u_k) \Rightarrow w\in N_G(u_{j-1})$.

Assume then that $w\in \cap _{k=j}^{|X|}N_G(u_k)$ and we deduce $w\in N_G(u_{j-1})$ applying again Fact 1 with $z = u_{j-1}$ and showing that (b’), hence (b), cannot hold. Indeed, supposing by contradiction that (b’) holds, it yields $N_C(u_{j-1}) \subseteq N_G(u)$. By this assumption and using the maximality of *C*, we deduce that $u \in X$ with the following arguments: $N_C(u)$ has to contain at least $N_C(u_{j-1})$, and hence there exists ${\tilde{k}}\ge j-1$ for which $u=u_{{\tilde{k}}}$ otherwise we could add the related edges.

Although $u \in X$ implies that we could contradictorily extend *C* to $C'$ by adding at least *e*, were $C'$ has the following list of neighbourhoods:

$$\begin{aligned} N_{C'}(u_k)&:= N_C(u_k)&\text { for }k \ne {\tilde{k}} \\ N_{C'}(u_k)&:= N_C(u_{k}) \cup \{w\}&\text { for }k = {\tilde{k}} \end{aligned}$$

and $C'$ is a chain graph since $N_C(u_{{\tilde{k}}}) \cup \{w\} \subseteq N_C(u_k)$ for all $k > {\tilde{k}} \ge j-1$ by $w\in \cap _{k=j}^{|X|}N_G(u_k)$ and the maximality of *C*. $\square$

Using Lemma 6 we can now prove the following result.

### **Theorem 4**

*Given a bipartite graph*$G=(U \cup W, E)$, *for any two edges*$e, e' \in E$, ${\mathscr {C}}_e \subseteq {\mathscr {C}}_{e'}$*if and only if*${\mathcal {M}}_e\supseteq {\mathcal {M}}_{e'}$.

### *Proof*

($\Rightarrow$) Given two edges $e, e' \in E$, suppose that ${\mathscr {C}}_e \subseteq {\mathscr {C}}_{e'}$, and assume on the contrary that there exists $f \in {\mathcal {M}}_{e'}$ and $f\not \in {\mathcal {M}}_{e}$. Then there exists a maximal chain subgraph $C'$ containing *e* and *f* (as they do not form a $2K_2$ in *G*) but not $e'$ ($f \in {\mathcal {M}}_{e'}$). Hence, $C' \in {\mathscr {C}}_e$ but $C' \notin {\mathscr {C}}_{e'}$, contradicting the assumption that ${\mathscr {C}}_e \subseteq {\mathscr {C}}_{e'}$ .

($\Leftarrow$) Suppose now ${\mathcal {M}}_e\supseteq {\mathcal {M}}_{e'}$. Let $C \in {\mathscr {C}}_{e}$. By definition, none of the edges of ${\mathcal {M}}_{e}$ appears in *C*. Hence, $e'$ does not form a $2K_2$ with any edge in *C* in the graph *G* (as ${\mathcal {M}}_e\supseteq {\mathcal {M}}_{e'}$). By Lemma 6$e' \in C$. Thus, ${\mathscr {C}}_e \subseteq {\mathscr {C}}_{e'}$. $\square$

Notice that, given an edge $e=(u, w) \in E$, $u \in U$ and $w \in W$, it is easy to determine the set ${\mathcal {M}}_e$. We just need to start from *E* and delete all edges that are incident either to *u* or to *w*, as well as all edges at distance 2 from *e* (that is all edges $e'=(u', w')$ such that either $u'$ is adjacent to *w* or $w'$ is adjacent to *u*). Checking whether ${\mathcal {M}}_e\supseteq {\mathcal {M}}_{e'}$ is also easy: it suffices to sort the edges in each set in lexicographic order, and then the inclusion of each pair can be checked in linear time in their size, that is in *O*(*m*). It is thus possible to enumerate in polynomial delay only those maximal chain subgraphs that contain at least one essential edge by slightly modifying Algorithm  1 as shown in the pseudo-code in Algorithm 2. 
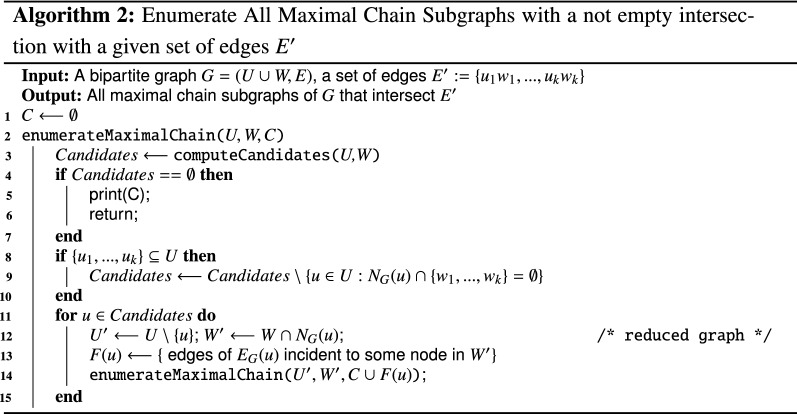


### **Theorem 5**

*Given a bipartite graph*$G=(U \cup W, E)$, *one can enumerate all its minimal chain subgraph covers*, *i.e.all the elements in*${\mathcal {S}}$, *in time*$O([({\mathcal {M}}+1)|{\mathcal {S}}|]^{\log (({\mathcal {M}}+1)|{\mathcal {S}}|)})$.

### *Proof*

We first construct the hypergraph ${\mathcal {H}}=(V,{\mathcal {E}})$ where $V:=E'$ is the set of essential edges of *G* and ${\mathcal {E}}:={\mathscr {C}}_{ess}$ is the set of maximal chain subgraphs of *G* that contain at least one essential edge. This takes time $O(n^2 m |{\mathscr {C}}_{ess}|)$. Applying then the algorithm given in [25], one can enumerate all minimal set covers of ${\mathcal {H}}$ (i.e. all minimal chain subgraph covers) in time $O((|{\mathcal {H}}|+|{\mathcal {S}}|)^{\log (|{\mathcal {H}}|+|{\mathcal {S}}|)})=O((|{\mathscr {C}}_{ess}|+|{\mathcal {S}}|)^{\log (|{\mathscr {C}}_{ess}|+|{\mathcal {S}}|)})$. The total running time is thus $O(n^2m |{\mathscr {C}}_{ess}|+ (|{\mathscr {C}}_{ess}|+|{\mathcal {S}}|)^{\log (|{\mathscr {C}}_{ess}|+|{\mathcal {S}}|)})$. Notice now that since by Lemma 5, every maximal chain subgraph in ${\mathscr {C}}_{ess}$ belongs to at least one minimal chain subgraph cover, we have that $|{\mathscr {C}}_{ess}|\le {\mathcal {M}}|{\mathcal {S}}|$. Finally, we obtain that the total running time is $O(n^2 m^2 |{\mathcal {S}}|+ ({\mathcal {M}}|{\mathcal {S}}|+|{\mathcal {S}}|)^{\log ({\mathcal {M}}|{\mathcal {S}}|+|{\mathcal {S}}|)}) = O([({\mathcal {M}}+1)|{\mathcal {S}}|]^{\log (({\mathcal {M}}+1)|{\mathcal {S}}|)})$. $\square$

## Chain graphs and interval orders

There is an interesting connection between chain graphs and interval orders. In this section, we look at the results we presented in this paper in the light of this relation, in particular related to the computation of the *interval order dimension* of a poset and the enumeration of *minimal interval order extensions* and *maximal interval order reductions* of bipartite posets. First, we briefly recall in this section these notions and this relation.

A *partially ordered set* (or in short *poset*) is a pair $(P,\le _P)$ where *P* is a set, called the *ground set*, and $\le _P\;\subseteq P \times P$ is a binary, reflexive, transitive and anti-symmetric relation on *P* referred to as *partial order on P*. A partial order is an *interval order on P* if there exist two functions $l,r: P \rightarrow \mathbb {R}$ such that $x \le _P y$ iff $r(x) \le l(y)$, while *P* is said to be a *total* or *linear* order iff either $x \le _P y$ or $y \le _P x$ for all $x,y \in P$. A partial order $Q = (Q,\le _Q)$ is said to *extendP* or *to be an extension ofP* if $x \le _P y$ implies $x \le _Q y$. A linear extension of *P* is an extension of *P* which is also a linear order. A *bipartite poset* is a poset $H = (U \cup V, \le _H)$ such that $\le _H\;\subseteq U \times V$.

The *interval order dimension* of a bipartite poset *H*, denoted by *Idim*(*H*), is the minimum number *k* of interval order extensions whose intersection gives *H*.

We can view *H* as a bipartite undirected graph, called the *comparability graph G(H) of H*, with node set $U \cup V$ and edge set given by $\{(u,v)\in E(G(H)): u \in U, v\in V, \text { and } u \le _H v\}$. We have that a bipartite poset is an interval order if and only if its comparability graph is a chain graph [20]. Hence each interval extension of *H* can be viewed as a chain graph (edge) completion of *G*(*H*), i.e. a chain graph with the same node set as *G*(*H*) which has *G*(*H*) as a subgraph. Thus *Idim*(*H*) coincides with the minimum number of chain graph completions of *G*(*H*) whose intersection gives *G*(*H*).

The *bipartite complement* of a bipartite graph $D = (U \cup V,E))$ is the graph $B(D) = (U \cup V, E')$ where $E'$ are all the non-edges of *D* across the two partitions.

Now, if *C* is a chain subgraph of *G*(*H*), also its bipartite complement is a chain graph (as the bipartite complement of a $2K_2$ is a $2K_2$). Then we have that *Idim*(*H*) coincides with the size of a minimum chain subgraph cover of the bipartite complement of *G*(*H*), which is our second problem. All this is contained in the following result of [20] where, by abuse of notation, the bipartite complement of *G*(*H*) is denoted by *B*(*H*) (instead of *B*(*G*(*H*))) and the size of a minimum chain subgraph cover of *B*(*H*) is denoted as *ch*(*B*(*H*)):

### **Proposition 5**

(Lemma 4 in [20]) *LetHbe a bipartite poset. Then*$Idim(H) = ch(B(H))$.

From this, we obtain an interpretation of our results on the computation of the size of a minimum chain subgraph cover (see Theorem 3):

### **Corollary 2**

*Given a bipartite posetHwhereB*(*H*) *hasmedges, for all*$k\in \mathbb {N}^{*}$*and for all*$\varepsilon >0$, *we can check if*$Idim(H)\le k$*in time*$O^*((2+\epsilon )^{m})$.

In the same way, enumerating all the maximal chain subgraphs of a bipartite graph *G*(*H*) (i.e. our first problem) is equivalent to listing all the maximal interval order reductions of the bipartite order *H* and enumerating all maximal chain subgraphs of *B*(*H*) is equivalent to listing all minimal interval order extensions of *H*.

We can then interpret the results on the enumeration of maximal chain subgraphs (Propositions 2 and 3) in this context as follows:

### **Corollary 3**

*Let*$H = (U \cup V, \le _H)$*be a bipartite poset with*$n = |U| + |V|$*and*$|R_{\le _H}| - n = m$. *Then:*

1. *We can enumerate its maximal interval order reductions in polynomial time delay with a delay of*$O(\min \{|U|,|V|\}^2m)$.
2. *We can enumerate its minimal interval order extensions in polynomial time delay with a delay of*$O(\min \{|U|,|V|\}^3\cdot \max \{|U|,|V|\})$.

### *Proof*

The first result is a straightforward interpretation of Propositions 2 and 3 in this context, while the second result comes from the fact that we have to run our algorithm on *B*(*H*) instead of *G*(*H*), hence the number of edges goes from *m* to $|U|\cdot |V| - m$, hence $O(n^2(|U|\cdot |V| - m) \}) = O(n^2\cdot |U|\cdot |V|)$. Finally, in both results, we apply the observation that we can run our algorithm on the smaller of the two partitions substituting $n^2$ by $\min \{|U|,|V|\}^2$. $\square$

Observe that it is not surprising that enumerating minimal extensions is more complicated than enumerating maximal reductions as it happens in [15] for counting minimal extensions and maximal reductions of N-free orders (i.e. the posets $(P,\le _P)$ such that there does not exist $x,y,z,w \in P$ with $x \le _P y$, $x \le _P w$ and $z \le _P w$).

Furthermore, recall that for general posets *P*, in [15] it was already proved that the number of minimal interval order extensions and maximal reductions can be computed polynomially in their number, but the proposed dynamic programming algorithm requires an exponential space to prevent duplications by storing all the already found solutions and comparing the new solution with them, differently from the Algorithm 1 we proposed.

## A case study

**Fig. 3:**
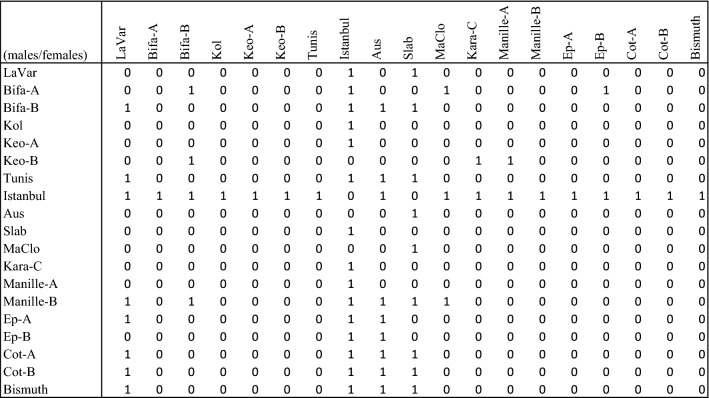
The *Culex pipiens* dataset in [6]. Rows represent the males and the columns the females. A value of 1 in the cell [*i*: *j*] represents an incompatibility between male *i* and female *j*

**Fig. 4:**
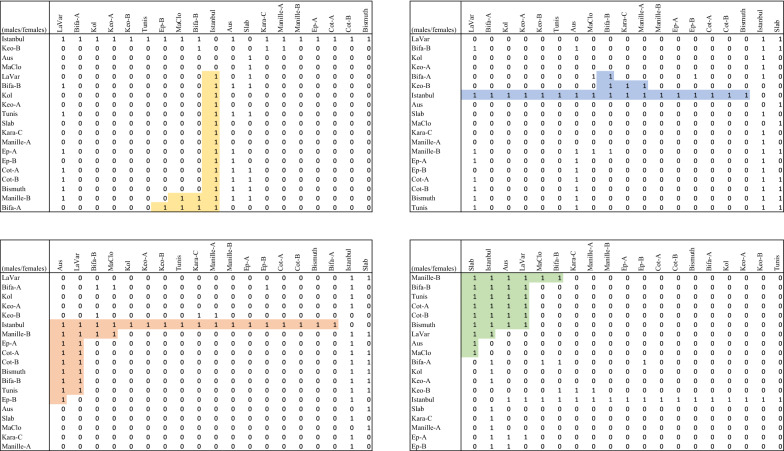
The *Culex pipiens* dataset in [6]. A minimum chain cover for the graph *G*, constituted by four chain subgraphs

In this section we show an application of our methods to a real dataset. We implemented Algorithm 1 and ran it on the graph *G* representing the CI of *Wolbachia* in *Culex pipiens* [6], stored by means of the incidence matrix in Fig. 3. The code is available at https://github.com/sinaimeri/ChainEnumeration. On this dataset, the algorithm to list all the maximal chains took only 1 second on a single core of a 6-core MacBook Pro 2.2 GHz i7. The maximal chain subgraphs of *G* came out to be 16, and by doing a simple exhaustive search algorithm, we found a chain cover of *G* constituted by 4 chain subgraphs (represented in Fig. 4 in colours on the matrix storing *G*, where rows and columns have been conveniently permuted in order to highlight the triangular shapes of chain subgraphs, that are called *quantitative shapes* in [6]). Notice that this cover is minimum since it is known [18] that the size of a maximum induced subgraph is upper bounded by the size of a minimum chain cover, and it is not difficult to find by hand a maximum induced subgraph of *G* with 4 edges (e.g. edges connecting Bifa-A and Istanbul, Keo-B and Bifa-B, Istanbul and Aus, and Aus and Slab, all corresponding to 1s on the incident matrix); this improves the result claimed in [6] (page E19) that 5 chain subgraphs would be necessary.

**Fig. 5:**
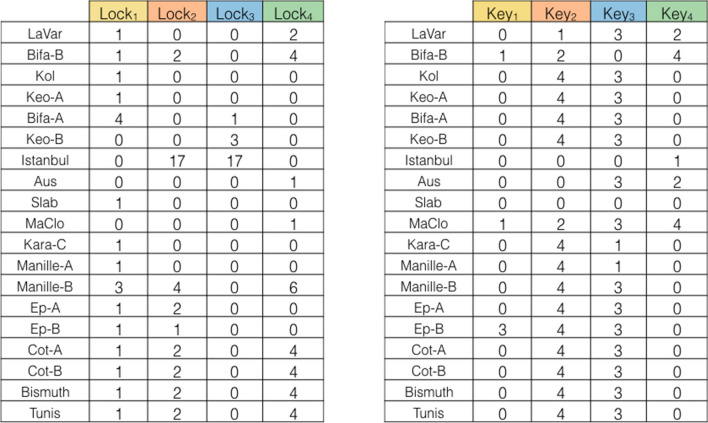
The Lock and Key matrices for the *Culex pipiens* dataset in [6]. The Lock matrix and the Key matrix resulting from the solution of the quantitative model. In each cell the relative amount of lock and key molecules is indicated. Notice that a value 0 indicate that no molecule is inferred from the analysis. Each color symbolizes a single Lock-Key pair

In Fig. 5, we show the corresponding matrices Lock and Key of solution. For each male (female) individual the amount of lock (key) molecules is indicated. Notice that a value 0 indicates that no lock or key molecule is inferred from the analysis. Each colour symbolizes a single Lock-Key pair. The values of the quantities are only indicative. Indeed, Lemma 1 provides a possible assignment that explains the incompatibilities but what is important are not the values themselves but the relative order among them. Thus, for instance we can safely substitute the value 17 in the table by 5 and the relative order will not change.

It is also interesting to observe that the solution provided in Fig. 5 is significantly different from the one in [6]. For instance, in [6] the Lock matrices inferred under the quantitative model contain many more empty cells than the Key matrices. This is clearly not the case in the solution we presented, where both Lock and Key matrices have roughly the same number of non empty cells, indicating a more uniform level of infection in the population considered. In our solution, each male or female individual is infected by 2 or 3 strains of *Wolbachia*.

## Conclusions and open problems

In this paper, we studied the problem of finding the minimum number of different Lock/Key molecules that explains the CI for the observed data. This problem was already modelled as finding the minimum number of chain subgraphs that cover the edges of a given bipartite graph [6, 9].

Motivated by this, we studied different problems related to maximal chain subgraphs and chain subgraph covers in bipartite graphs. First, for the NP-hard problem of finding the minimum number of chain subgraphs in a bipartite graph, we provided an exponential algorithm with a non trivial complexity. Although we improved the complexity in theory, a simple implementation may not be efficient for large and dense graphs. A future direction would be to use this algorithm as a base for more efficient implementations that are fast in practice.

Second, for the problem of enumerating all minimal chain subgraph covers of a bipartite graph, we showed that it can be solved in quasi-polynomial time. It remains an open problem to understand whether it is possible to enumerate the minimal chain covers of a graph in polynomial delay.

Interestingly, in order to solve the above problems, we considered also the problem of enumerating all the maximal chain subgraphs of a bipartite graph and improved on the current results in the literature for the latter. It is worth exploring the different nature of the problems considered here in the case where we deal with an hereditary property (induced chain subgraphs) instead of a non-hereditary one (edge induced chain subgraphs).

Finally, in this paper we assumed the data are correct and complete. It is certainly interesting to deal with the case of missing data.
